# Tissue-Specific Chemerin in Atherosclerosis

**DOI:** 10.3390/biom16091327

**Published:** 2026-09-11

**Authors:** Muyun Chen, Yuxin Li, Linling Feng, Longhua Liu

**Affiliations:** School of Exercise and Health, Shanghai University of Sport, Shanghai 200438, China

**Keywords:** chemerin, atherosclerosis, cardiovascular diseases

## Abstract

Atherosclerotic cardiovascular disease (ASCVD) remains the leading cause of disability-adjusted life years (DALYs) and all-cause mortality worldwide, imposing an increasing global health burden. Atherosclerosis (AS) is a complex multifactorial disease, and the concept of organ crosstalk has provided a new perspective for understanding its pathogenesis. Although circulating chemerin levels are closely associated with the development and adverse prognosis of ASCVD, the functional heterogeneity and regulatory characteristics of chemerin derived from different tissues have not been systematically investigated. This review elucidates the tissue-specific roles of chemerin in AS from both endocrine and paracrine perspectives, with a focus on chemerin derived from the liver, white adipose tissue, perivascular adipose tissue, and epicardial adipose tissue. Endocrine chemerin primarily contributes to systemic metabolic dysfunction and endothelial injury, whereas locally produced chemerin regulates vascular smooth muscle cell remodeling and vascular inflammation through paracrine signaling. Collectively, endocrine and paracrine chemerin constitute a dual regulatory network that links metabolic abnormalities with vascular remodeling during AS progression.

## 1. Introduction

Atherosclerotic cardiovascular disease (ASCVD) comprises a group of cardiovascular disorders caused by Atherosclerosis (AS), including ischemic heart disease (IHD), ischemic stroke, and peripheral arterial disease. According to the Global Burden of Disease 2023 study, cardiovascular diseases remain the leading cause of disability-adjusted life years (DALYs) and all-cause mortality worldwide, with IHD accounting for the largest proportion of disease burden among all cardiovascular subtypes [1,2]. Despite substantial advances in pharmacological therapy and revascularization techniques, most patients with advanced ASCVD still require percutaneous coronary intervention or surgical revascularization, imposing considerable health and socioeconomic burdens [3,4,5]. Therefore, a better understanding of the molecular mechanisms underlying AS is essential for the development of effective preventive and therapeutic strategies.

AS is a chronic progressive disease characterized by the accumulation of lipids, inflammatory cells, vascular smooth muscle cells (VSMCs), and necrotic debris within the subendothelial space [6,7]. Owing to its multifactorial nature, numerous hypotheses have been proposed to explain its pathogenesis. In recent years, advances in multi-omics technologies have shifted the understanding of AS from a vascular-centered disease toward a systemic disorder driven by inter-organ communication. Increasing evidence suggests that metabolic organs including adipose tissues, and the cardiovascular system establish a dynamic signaling network through endocrine and paracrine mediators, thereby collectively regulating vascular homeostasis and atherosclerotic progression [8,9]. Chemerin was initially identified as an inflammatory chemokine that recruits antigen-presenting cells (APCs), thereby participating in the early recruitment of immune cells. It was not until 2007 that chemerin was discovered in white adipose tissue and identified as an adipokine that regulates adipogenesis and adipocyte metabolism [10]. As a pleiotropic secreted protein, chemerin exhibits a significant role in driving cardiovascular pathology. Acting as a critical regulator of glucose and lipid homeostasis, it has been reported to strongly correlate with systemic inflammatory markers and the metabolic syndrome [11,12]. Currently, chemerin has emerged as an important cardiovascular biomarker. Numerous clinical and prospective cohort studies have indicated that circulating chemerin levels are closely associated with the incidence and poor prognosis of cardiovascular diseases (CVDs) [13,14]. Although the pro-atherosclerotic effects of chemerin have been extensively reported, there is currently a lack of systematic analyses and summaries of chemerin’s functions derived from different tissue sources. This review aims to systematically categorize the functions of tissue-specific chemerin in cardiovascular pathology, differentiate its distinct biological roles mediated via endocrine and paracrine pathways, and summarize its common mechanisms in promoting AS and its multi-organ synergistic regulatory effects, which may provide novel theoretical perspectives for precision-targeted therapy of ASCVD.

## 2. Search Strategy

We systematically searched the PubMed and Web of Science databases for English-language publications from 2003 (when chemerin was first named) to 2026. The search topics included *RARRES2*, coronary artery disease, atherosclerosis, inflammation, metabolic diseases, and related receptors. Keywords conforming to the Medical Subject Headings criteria were used. Particular emphasis was placed on studies published within the past 10 years. All articles that met the methodological criteria and were considered relevant to the scope of this review were included. The subtopics of this review were determined through discussion among the authors. The literature was independently reviewed and organized by the four authors, who confirmed that the included articles met the predefined search criteria and were suitable for the analysis and evaluation of the objectives of this review.

## 3. Structure and Isoforms of Chemerin

The *RARRES2* gene encodes human chemerin and is initially translated as a 163-amino-acid protein preprochemerin [15]. Preprochemerin comprises a 20-amino-acid N-terminal hydrophobic signal peptide, a 137-amino-acid cystatin-like fold domain, and a 6-amino-acid C-terminal propeptide. Cleavage of the N-terminal signal peptide yields prochemerin (huChem163S), which possesses low biological activity. Subsequently, the C-terminus of huChem163S is proteolytically cleaved by various specific proteases involved in coagulation, fibrinolysis, and inflammatory cascades [16,17], thereby generating multiple highly active chemerin variants, such as huChem156, huChem157, and huChem158. Further over-hydrolysis can degrade these active isoforms into shorter fragments; proteins with fewer than 155 amino acids may typically function as inactive or low-activity. The N-terminus of chemerin is critical for stabilizing its receptor binding. Chemotaxis assays using hCMKLR1/L1.2-engineered cells demonstrated that the full-length activity of each chemerin variant surpasses that of its corresponding C-terminal short-chain peptide, a phenomenon particularly evident in the low-bioactivity variant huChem155 [18]. Cryo-EM further confirmed that the N-terminus of chemerin interacts with chemokine-like receptor 1 (CMKLR1), thereby stabilizing the chemerin-CMKLR1 complex and ensuring the efficient insertion of chemerin’s C-terminus into the orthosteric binding pocket of CMKLR1 to activate the receptor and trigger downstream signaling [19]. The C-terminal sequence of chemerin and its proteolytic cleavage sites are conserved. Different C-terminal peptides induce distinct signaling outcomes.

The C-terminus of huChem163 can be cleaved at different sites by various proteases, generating isoforms with distinct biological activities. HuChem158 can be generated from huChem163 by cleavage with plasma factor XIa, with huChem162 serving as an intermediate product. However, its activity is similar to that of huChem163, suggesting that huChem162 may have limited intrinsic biological activity [16]. In addition, plasmin and tryptase can cleave huChem163 to generate huChem158 [20]. The C-terminal lysine of huChem158 can be removed by carboxypeptidase B and carboxypeptidase N, generating huChem157 [16]. Cathepsin L, cathepsin K, and elastase can also directly process huChem163 into huChem157 [21,22]. Staphopain B, a protease secreted by Staphylococcus aureus, can likewise mediate the generation of huChem157; however, whether a similar protease exists under physiological conditions in humans remains to be determined [23]. HuChem156 is derived from huChem163 through cleavage by kallikrein 7, cathepsin G, and chymase [24,25,26]. Tryptase, proteinase 3, and elastase can cleave huChem163 to generate huChem155 [20,27]. Notably, variants shorter than 155 amino acids are considered inactive. Chymase, cathepsin L, cathepsin K, and elastase have been reported to further cleave chemerin, thereby promoting its inactivation [28].

The mouse chemerin protein is one amino acid shorter than the human counterpart, and species-specific functional differences exist among certain isoforms. For instance, huChem-157 and its mouse counterpart, muChem-156, represent the highest-activity variants in their respective species. Although the potency of muChem-155 is comparable to that of muChem-156, the human homolog huChem-156 exhibits a far lower potency than huChem-157 [29]. The composition and abundance of chemerin depend on the organism’s physiological and pathological status. In normal human blood circulation, huChem163 is predominant, and diet, sex, and age do not affect the distribution of chemerin isoforms in the body [30]. Under pathological conditions, huChem163 in localized tissue fluids is converted into shorter truncated variants, such as huChem-156, huChem-157, and huChem-158, which are highly abundant [31]. Both aging and obesity are associated with elevated total chemerin levels [29,30,31]. Enzyme-linked immunosorbent assays (ELISAs) showed that 12-week HFD-induced total chemerin elevation was dominated by muChem157 and muChem156, while muChem162 contributed most after 26-week HFD. In addition, high muChem162 was also found in aged mouse blood [29]. Conversely, the synthetic short peptide chemerin-9 has been found to exert therapeutic effects in memory impairment [32,33], AS [34], abdominal aortic aneurysm [35,36], and pancreatogenic diabetes [37]. Studies have shown that cathepsin S, a protease produced by activated macrophages, can cleave murine prochemerin to generate the anti-inflammatory isoform Chemerin-15 (A140–A154) [38]. Chemerin-15 inhibits the secretion of pro-inflammatory mediators by macrophages and reduces neutrophil recruitment [39,40]. Its anti-inflammatory effect is comparable to that of muChem154. Although Chemerin-15 lacks the key structural elements required for CMKLR1 activation, its anti-inflammatory effects are completely dependent on CMKLR1 [41]. These findings indicate that chemerin is not a single bioactive molecule; rather, selective cleavage of its C-terminal peptide by different proteases generates multiple isoforms with distinct functions, thereby mediating diverse biological effects.

## 4. Transcriptional Regulation of Chemerin Expression

The constitutive expression of *Rarres2* is influenced by the methylation of its promoter region located at −252 to +258 bp [42]. Beyond this baseline, metabolic and inflammatory signals can further modulate its expression by binding transcription factors, such as peroxisome proliferator-activated receptor γ (PPARγ) [43] and farnesoid x receptor (FXR) [44], to functional response elements on its promoter.

PPARγ belongs to the ligand-activated transcription factor superfamily of nuclear hormone receptors. PPARγ directly affects gene expression by binding to the proximal direct repeat 1 (pDR1) element within the −61 to −49 bp region of the mouse *Rarres2* promoter. The PPARγ agonist rosiglitazone significantly upregulates *Rarres2* expression in mouse bone marrow, adipose tissue, and the trophoblast cell line HTR8-SVneo [43,45]. In contrast, using troglitazone to activate PPARγ in human mature adipocytes cultured in vitro results in an 80% decline in chemerin secretion [46]. Similarly, a significant reduction in chemerin levels was observed following pioglitazone treatment in patients with type 2 diabetes [47]. This bidirectional regulatory phenomenon may result from modifications of PPARγ co-regulators driven by stress and inflammatory microenvironments.

FXR is a nuclear receptor that regulates bile acid levels, and the liver is the primary organ of its expression. In vitro experiments revealed that the FXR agonist GW4064 enhances chemerin expression in HepG2 cells and primary hepatocytes, an effect that disappears upon FXR knockout. Furthermore, the FXR response element (FXRE) was identified within the −258 to −90 bp region of the *Rarres2* promoter. Thus, FXR exerts a direct transcriptional regulatory role on *Rarres2*, at least in the liver [44]. In addition, many transcription factors, including nuclear factor kappa-B (NF-κB), activator protein 1 (AP-1), c-Jun/c-Fos, interferon regulatory factor 1 (IRF1), or signal transducer and activator of transcription 1 (STAT1), responding to pro-inflammatory cytokines, might regulate *Rarres2* [48]. However, this regulation exhibits cell-type specificity. Studies have found that tumor necrosis factor-alpha (TNF-α) treatment significantly increases chemerin secretion in mouse adipocytes, whereas hepatocytes do not show the same benefit [29]. Similar outcomes have been observed for interleukin 1β (IL-1β) and oncostatin M [42]. However, within the ChIP-Atlas database, no transcription factors were found capable of binding to the -735 to +258 bp region of the *Rarres2* promoter in either adipocytes or hepatocytes [42]. This indicates that inflammation-responsive transcription factors are highly likely to influence chemerin expression through alternative mechanisms or cis-regulatory elements.

## 5. Chemerin Receptors

Following the proteolytic cleavage of its C-terminus, chemerin exerts its biological functions by binding to the receptors c-c motif chemokine receptor-like 2 (CCRL2), CMKLR1, or g protein-coupled receptor 1 (GPR1). CMKLR1 is a classic class-A G protein-coupled receptor (GPCR) that binds chemerin. CMKLR1 is expressed in a variety of cells involved in inflammatory responses, including monocytes, dendritic cells, and natural killer (NK) cells [49]. The activation of CMKLR1 drives lipid metabolism and inflammatory responses via G-protein signaling pathways, which include triggering intracellular calcium release, reducing cyclic adenosine monophosphate (cAMP), phosphorylating extracellular signal-regulated kinase (ERK) and mitogen-activated protein kinase (MAPK), activating RhoA, recruiting β-arrestin2, and inducing receptor internalization [15,50,51]. GPR1, conversely, is an atypical GPCR that participates in physiological activities through β-arrestin2-mediated signaling pathways. Under lipid overload conditions, GPR1 clears the antagonistic chemerin produced by adipose tissue via β-arrestin2-mediated internalization, thereby creating favorable conditions for CMKLR1 to activate G-protein signaling and promote lipolysis, which ultimately reduces triglyceride accumulation [52]. CCRL2 belongs to the atypical chemokine receptor family and helps regulate localized chemerin levels and bioactivity [53,54]. Chemerin is incapable of triggering CCRL2 internalization, chemotaxis, or calcium mobilization; instead, CCRL2 serves to present the enriched chemerin to the other two signaling receptors to exert its functions [55]. Furthermore, chemerin can act independently of binding to CMKLR1, activating monocyte β-arrestin2 through its intrinsic PDI-like activity [56].

## 6. Chemerin in Different Tissues and Its Biological Functions

**Figure 1:**
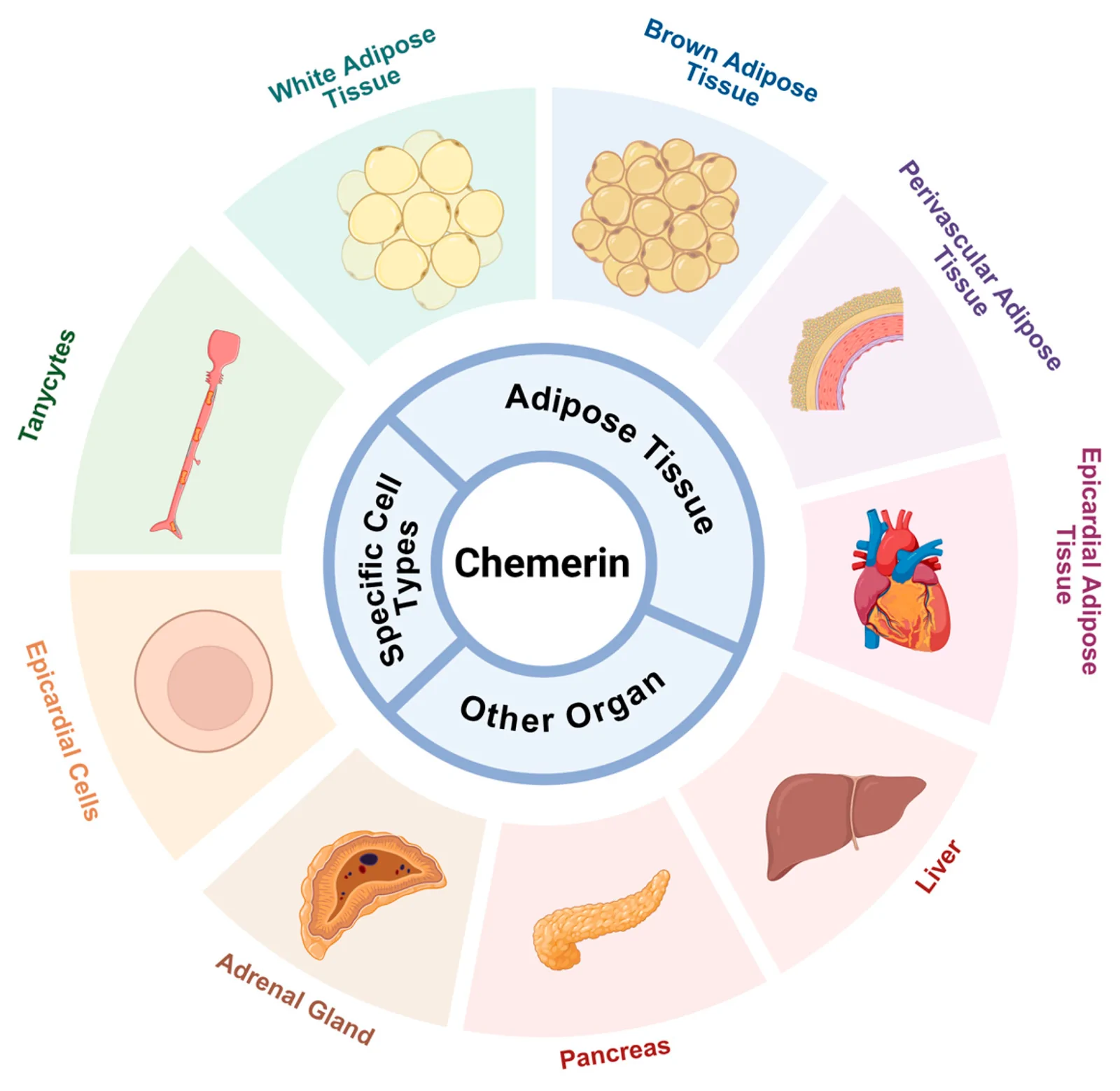
Different tissue origins of chemerin.

AS is not merely an independent local vascular lesion but a systemic metabolic disorder involving multiple organs. Distal metabolic organs can participate in and regulate cardiovascular homeostasis and pathological remodeling through paracrine and endocrine networks. Chemerin is expressed in multiple human organs and tissues, with the highest levels observed in the liver and white adipose tissue. Certain specific cell types, including epicardial and elongation cells, can also secrete chemerin. In addition, epithelial cells, endothelial cells, fibroblasts, chondrocytes, and platelets have all been reported to exhibit chemerin immunoreactivity [57]. (Figure 1) However, chemerin activation differs among tissues. Tissues with high levels of secreted chemerin do not necessarily show high expression of its receptors CMKLR1 and GPR1; conversely, tissues with a high expression of CMKLR1 and GPR1 may not necessarily secrete chemerin. This suggests that chemerin secreted by certain tissues may circulate via endocrine pathways to tissues with low or absent chemerin expression, where it undergoes cleavage and activation, or acts independently of the circulation to regulate the properties of adjacent tissues through paracrine mechanisms.

### 6.1. Systemic Endocrine Sources of Chemerin

Hepatic dysfunction has long been recognized as an independent or secondary risk factor for CVDs. Increasing evidence suggests a bidirectional physiological crosstalk between the liver and the heart. Metabolic dysfunction-associated steatotic liver disease (MASLD) is an often-unappreciated independent risk factor for ASCVD [58,59,60]. Liver-derived secretory proteins are key mediators linking hepatic metabolism to systemic homeostasis, particularly in cardiometabolic diseases. Chemerin is highly expressed in the liver [17], including hepatocytes, hepatic stellate cells, and endothelial cells, but not in Kupffer cells.

High levels of chemerin are detected in both human liver tissue and primary hepatocytes [61]. Antisense oligonucleotide (ASO)-mediated liver-specific inhibition of chemerin expression in rats significantly reduces circulating chemerin levels [62]. Similarly, adeno-associated virus (AAV)-mediated hepatic overexpression of human chemerin in *Ldlr*^−/−^ mice also increases circulating chemerin levels [63].

Adipose tissue is also considered a major source of chemerin secretion. In the hepatic vascular system, metabolites from visceral adipose tissue primarily return to the liver via the portal vein. However, significant concentration differences between the hepatic and portal veins in patients with cirrhosis (higher levels in the hepatic vein, with no difference between the portal and peripheral veins) suggest that hepatic contribution to circulating chemerin may exceed that of adipose tissue [64]. Excessive lipid accumulation in the liver stimulates hepatic chemerin synthesis. Chemeirn expression is increased in HFD-fed mice, *ob/ob* mice, *db/db* mice, and patients with human fatty liver disease [65,66]. Similar changes are also observed in Paigen diet- and methionine choline deficient (MCD)-induced metabolic dysfunction-associated steatohepatitis (MASH) mouse models, as well as in human MASH liver tissues [66,67,68]. Liver-specific chemerin knockout reduces HFD-induced increases in triglycerides, total cholesterol, and low-density lipoprotein cholesterol levels in diet-induced obese mice, and alleviates hepatic injury and systemic inflammation [69]. These findings suggest that, under pathological conditions, large amounts of chemerin entering the circulation act as endocrine factors broadly affecting peripheral tissues, thereby exacerbating systemic metabolic disorders and promoting the development of AS.

White adipose tissue (WAT), as an important endocrine organ, secretes multiple adipokines to regulate systemic metabolism [70,71]. Similar to the liver, chemerin is also highly expressed in WAT. Chemerin participates in the regulation of multiple adipose tissue functions. In addition to the high expression of chemerin and its receptors in WAT, adipocytes themselves express elastase- and trypsin-like proteases required for the cleavage of chemerin precursors [30,72]. Moreover, immune cell infiltration induced by obesity further increases local cathepsin levels in adipose tissue [73,74,75]. These findings support that active chemerin variants can be generated locally within adipose tissue and exert autocrine/paracrine effects [29].

Inflammation is considered a central trigger of AS. Chemerin-mediated immune cell recruitment, along with its interaction with local damage-associated molecular patterns, further amplifies adipose tissue inflammation. Under systemic metabolic dysregulation, adipose tissue secretes large amounts of chemerin, recruiting plasmacytoid dendritic cells (pDCs), myeloid dendritic cells (mDCs), macrophages, and NK cells from the circulation into adipose depots [57,76]. Stress or necrosis of adipocytes leads to substantial release of high-mobility group box protein 1 (HMGB1) [77], which binds to toll-like receptor 9 (TLR9) to activate pDCs and initiate type I interferon responses [78,79]. In addition, dendritic cells in adipose tissue highly express major histocompatibility complex class II molecules and participate in antigen presentation, thereby activating CD4+ T cells and promoting interferon-gamma secretion, which drives visceral adipose inflammation [78,80]. NK cells further release large amounts of TNF-α, altering adipose tissue macrophage function and metabolic homeostasis [81,82]. Current evidence strongly suggests that chemerin likely plays a key mediating role in adipose tissue inflammatory responses.

The primary function of white adipocytes is dynamic lipid storage and mobilization [83]. Chemerin has been shown to promote the differentiation of preadipocytes into mature adipocytes. Conversely, lipolysis is suppressed in *Rarres2*^−/−^ mice [84]. Low concentrations of chemerin transiently and reversibly stimulate ERK1/2 phosphorylation [10], high concentrations reduce intracellular cAMP levels and exert anti-lipolytic effects [85]. However, in obesity, elevated circulating chemerin may further enhance lipid accumulation via its anti-lipolytic action, leading to tissue hypoxia, fibrosis, and chronic inflammation, thereby contributing to cardiometabolic disorders, including cardiovascular disease [86,87].

On the other hand, brown and beige adipose tissues dissipate energy through thermogenesis. Chemerin is expressed during brown adipocyte differentiation and plays a key role in energy metabolism by regulating thermogenesis in brown adipose tissue [88]. The reduction in brown and beige adipose tissues is closely associated with obesity [89]. Cold exposure decreases chemerin expression in brown adipose tissue (BAT) [90], whereas high-fat diets increase its expression. However, due to the lack of correlation with circulating chemerin levels, chemerin produced in this context is unlikely to act through an endocrine mechanism [90].

#### 6.1.1. Systemic Netabolic Dysregulation

**Figure 2:**
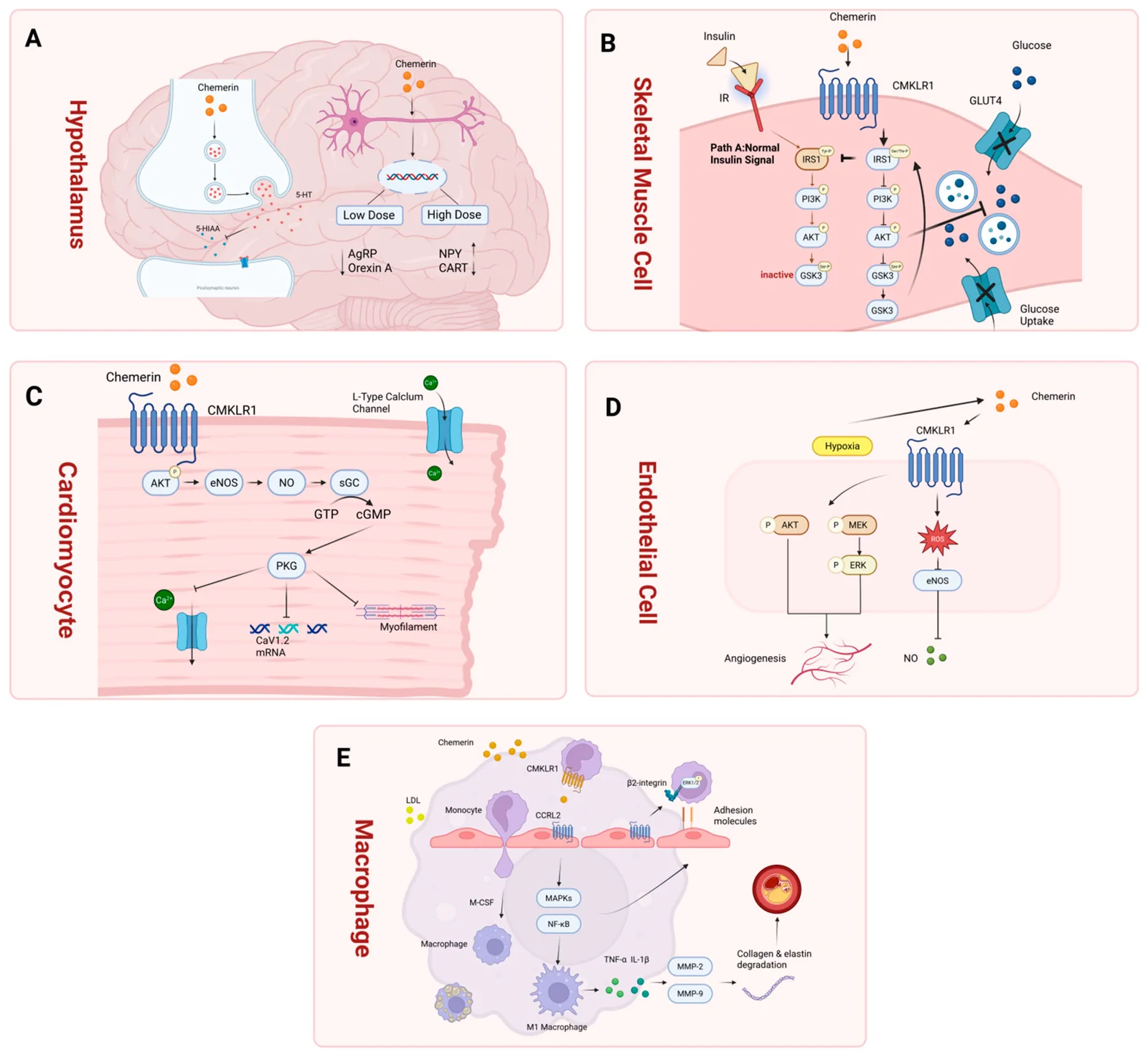
Role of endocrine-derived chemerin in regulating AS. (**A**) Chemerin affects systemic metabolic homeostasis through abnormal regulation of hypothalamic feeding behavior. (**B**) Chemerin inhibits the IRS1/PI3K/AKT signaling cascade in skeletal muscle, blocking GLUT4 translocation to the cell membrane and inducing insulin resistance. (**C**) Chemerin attenuates myocardial contractility by impairing the permeability of L-type calcium channels on the cardiomyocyte membrane through the PI3K/Akt/eNOS signaling cascade, reducing intracellular calcium influx. This negative inotropic effect, together with insulin resistance, contributes to the deterioration of cardiac function. (**D**) In endothelial cells, chemerin activates the NF-κB and MAPK pathways, increasing adhesion molecule expression, while ROS-mediated inhibition of eNOS reduces NO production. Hypoxia-induced upregulation of chemerin further regulates AKT/ERK signaling to promote angiogenesis. (**E**) Chemerin promotes monocyte recruitment through two complementary mechanisms. On the one hand, chemerin activates the CMKLR1-mediated NF-κB and MAPK signaling pathways in endothelial cells, leading to the upregulation of adhesion molecules and enhanced monocyte–endothelial adhesion. On the other hand, chemerin binds to endothelial CCRL2, which either facilitates the recruitment of CMKLR1-positive monocytes or promotes monocyte recruitment through chemerin-dependent activation of β2 integrins. In addition, chemerin is involved in inducing M1 polarization of macrophages, leading to the release of pro-inflammatory cytokines and elevated expression of MMPs, which promote the degradation of collagen and elastin, then increase plaque instability.

Chemerin is closely associated with metabolic disorders and inflammatory responses. Circulating chemerin levels are strongly correlated with metabolic syndrome [66,91]. Chemerin, secreted by the liver and adipose tissue, not only acts in an autocrine manner but also exerts endocrine effects on distant tissues, inducing local metabolic abnormalities and generating a cascade amplification effect that drives systemic metabolic dysregulation. Peripheral administration of chemerin reduces hypothalamic agouti-related peptide and orexin A expression while promoting serotonin synthesis and release [92], indicating that distal target tissues are responsive to circulating chemerin (Figure 2A). Insulin is a key hormone regulating systemic metabolic homeostasis, and insulin resistance is a central driver of AS. Skeletal muscle accounts for up to 80% of postprandial glucose uptake [93]. Although skeletal muscle does not express chemerin, it highly expresses its receptor CMKLR1. Chemerin impairs glucose uptake in skeletal muscle cells by modulating the phosphorylation status of insulin receptor substrate 1, protein kinase B (Akt), and glycogen synthase kinase-3, thereby inducing insulin resistance [46]. (Figure 2B) Similarly, chemerin activates the ERK signaling pathway and disrupts insulin signaling in cardiomyocytes, leading to myocardial insulin resistance [94]. Acute chemerin infusion induces negative inotropic effects in rat hearts via NO signaling activation and dysregulation of calcium homeostasis through downregulation of CaV1.2 expression [95]. (Figure 2C) However, in pancreatic β-cells, chemerin modulates glucose-stimulated insulin secretion [96]. *Rarres2*^−/−^, *Cmklr1*^−/−^, and *Gpr1*^−/−^ mice exhibit impaired glucose tolerance, which persists under both high-fat and normal diets [97,98,99,100]. These findings indicate that chemerin plays a dual role in metabolic regulation. The average plasma chemerin concentration in healthy lean men is approximately 81 ng/mL, and 87 ng/mL in healthy lean women [101]. In vitro, concentrations as low as 10 ng/mL are sufficient to elicit responses in cardiomyocytes, indicating that chemerin’s dual effects occur under both physiological and pathological conditions.

#### 6.1.2. Vascular Endothelial Cells

Although endothelial cells form a single-cell layer in all organs, vascular endothelium represents a highly dynamic and interactive system. Endothelial dysfunction involves maladaptive alterations in hemostasis and thrombosis regulation, vascular tone and redox balance, and inflammatory responses [102]. Endothelial cells are directly exposed to circulating blood and are therefore highly susceptible to chemerin (Figure 2D).

Chemerin induces NOX-derived reactive oxygen species (ROS) production, inhibits eNOS activation, and reduces nitric oxide (NO) production [103], thereby impairing barrier integrity and promoting LDL infiltration [104]. Hypoxia is a major driver of intraplaque neovascularization. Under hypoxic conditions, TNF-α activates ERK signaling, promoting the binding of transcription factor SP1 to *Rarres2*, thereby increasing its expression [105]. Chemerin promotes endothelial tube formation via CMKLR1-mediated activation of AKT and ERK pathways, with effects comparable to VEGF [106,107]. Endothelial progenitor cell adhesion and migration are also enhanced by chemerin through the p38 mitogen-activated protein kinase (MAPK) pathway [108]. Newly formed intraplaque vessels exhibit loose endothelial junctions and lack pericyte coverage, making them prone to leakage and intraplaque hemorrhage, which contributes to plaque instability [109]. These findings suggest that chemerin may contribute to plaque destabilization by regulating angiogenesis.

#### 6.1.3. Macrophage

(Figure 2E) As a chemokine, chemerin enhances monocyte–endothelial adhesion by activating CMKLR1-mediated NF-κB and MAPK signaling pathways and upregulating E-selectin, vascular cell adhesion molecule-1 (VCAM-1), and intercellular adhesion molecule-1 (ICAM-1) [110]. CCRL2 functions as a non-signaling chemerin-binding receptor that immobilizes chemerin on the endothelial surface by binding its N-terminal domain, thereby exposing the C-terminal bioactive region for interaction with CMKLR1 on circulating monocytes [53]. Notably, chemerin-dependent monocyte adhesion can also occur independently of CMKLR1 signaling. Disturbed flow has been shown to upregulate endothelial CCRL2 expression and promote chemerin-dependent activation of β2 integrins on monocytes [56]. Recruited monocytes migrate into the intima and differentiate into macrophages under macrophage colony-stimulating factor stimulation. These macrophages internalize oxLDL via scavenger receptors, eventually transforming into foam cells. Foam cells are dysfunctional, cholesterol-loaded macrophages. The uptake of circulating cholesterol crystals by macrophages activates the inflammasome, leading to the release of pro-inflammatory cytokines [111]. Pro-inflammatory cytokines further downregulate cholesterol efflux genes such as ATP-binding cassette transporter A1 and ATP-binding cassette sub-family G member 1 in macrophages, accelerating foam cell formation [112]. Notably, chemerin immunoreactivity has been observed in foam cells within atherosclerotic lesions [113]. Chemerin can activate the NF-κB pathway in endothelial cells and promote inflammatory responses. In the context of chronic inflammation, chemerin promotes the differentiation of macrophages toward the pro-inflammatory M1 phenotype [114]. Multiple cytokines secreted by macrophages, including TNF-α and IL-1β, can stimulate the production and secretion of matrix metalloproteinases (MMPs) [115]. MMPs are endopeptidases responsible for tissue remodeling and the degradation of extracellular matrix proteins and can degrade various components of the extracellular matrix that are essential for maintaining vascular wall integrity. Among these, MMP-2 and MMP-9 are gelatinases that are commonly expressed in vascular tissues and have been reported to be associated with cardiovascular diseases, including acute coronary syndrome and abdominal aortic aneurysm [111]. Both are key proteases involved in the degradation of the fibrous cap and the development of plaque instability. Recent studies have also indicated that MMP-25 is associated with pathological vascular remodeling. MMP-25 deficiency can attenuate the development and progression of AS, enhance plaque stability, and downregulate NF-κB activation, chemokine expression, and MMP-9 activity [116].

In addition, chemerin also recruits pDCs to atherosclerotic lesions. Recruited pDCs promote AS by producing type I interferons, which enhance the cytotoxic activity of T cells within the plaque [117]. In addition, pDCs have been reported to recruit macrophages into the vessel wall and are associated with pro-inflammatory cytokine levels and macrophage content in atherosclerotic lesions [118,119].

### 6.2. Local Paracrine-Derived Chemerin

Perivascular adipose tissue (PVAT) refers to a specialized adipose tissue surrounding blood vessels. Structurally, it provides mechanical protection to vessels during the contraction of adjacent tissues. With advances in adipose tissue research, PVAT has been recognized to play an important role in vascular physiology and pathology via paracrine mechanisms due to its unique anatomical location. The area and volume of PVAT have been shown to positively correlate with atherosclerotic plaque burden and may serve as a potential marker of atherosclerotic morphology or stability [120,121].

PVAT exhibits heterogeneity across anatomical locations and species. In rodents, the thoracic aorta is surrounded by brown adipose tissue-like depots, whereas the abdominal aorta is surrounded by adipose tissue containing mixed white and brown adipocytes [122]. In mice, coronary arteries lack surrounding adipose tissue, whereas in other rodents and humans, coronary arteries are surrounded by adipose tissue [122]. PVAT differs functionally from WAT. Human coronary PVAT shows a lower adipocyte differentiation state compared with subcutaneous and visceral adipose tissue, accompanied by significantly increased pro-inflammatory cytokine secretion and enhanced inflammatory cell-recruitment capacity [123]. Transplantation experiments in *Apoe*^−/−^ mice demonstrate that mice receiving inflammatory visceral PVAT transplants are more prone to AS than those receiving non-inflammatory subcutaneous adipose tissue [124]. These findings indicate that PVAT can affect adjacent vessels via paracrine signaling.

Chemerin is highly expressed in PVAT, whereas its receptor CMKLR1 is highly expressed in vascular tissue. Liver-specific silencing of chemerin using antisense oligonucleotides (ASO) does not reduce blood pressure in hypertensive rats, whereas systemic knockout improves it, suggesting that circulating chemerin may play a limited role in vascular regulation. Histological studies show that chemerin infiltration in periaortic and pericoronary adipose tissue and foam cells correlates significantly with the severity of AS [113]. In *Apoe*^−/−^ mice, vascular wall chemerin levels increase with disease progression, accompanied by upregulation of p-p38 MAPK, NF-κB p65, proliferating cell nuclear antigen, phosphorylated c-Jun N-terminal kinase (p-JNK), and p-ERK1/2, whereas AAV-mediated systemic chemerin deletion reverses these changes [125]. Active chemerin derived from PVAT not only modulates the sympathetic nervous system [126] but also amplifies norepinephrine- and 5-hydroxytryptamine-induced vasoconstrictive signaling [127], leading to arterial contraction and promoting aortic stiffening.

Epicardial adipose tissue (EAT) is located between the myocardium and the visceral layer of the epicardium. As an adipose depot directly connected to the heart surface, EAT interacts closely with coronary arteries through paracrine signaling. Under pathological conditions, EAT undergoes remodeling toward a pro-inflammatory and pro-fibrotic phenotype, concentrating systemic adipose-driven inflammatory effects within the heart via paracrine secretion of inflammatory adipokines. Cross-sectional and imaging studies consistently show that EAT volume, density, and distribution are closely associated with coronary artery disease (CAD) [128,129,130]. Clinical studies have shown that in CAD patients, mRNA expression of chemerin, TNF-α, and IL-1β is significantly upregulated in EAT and positively correlated with disease severity, whereas circulating chemerin levels show no significant difference [131]. However, the functional role of EAT-derived chemerin remains insufficiently studied. Recent evidence indicates that epicardial cells sense pressure overload and act by secreting chemerin paracrinally to induce fibroblast activation and myocardial fibrosis [132].

The concept of bone marrow adipose tissue (BMAT) has received increasing attention. Similar to other adipose tissues, BMAT can influence the bone marrow microenvironment through the secretion of adipokines [133]. Chemerin, acting within the bone marrow, is primarily secreted by BMAT [134]. Chemerin binds to CMKLR1 on hematopoietic stem cells (HSCs), thereby inducing osteoclast differentiation and bone matrix resorption [135]. The use of a chemerin-neutralizing antibody was found to completely block the osteoclast differentiation of HSCs, whereas the addition of exogenous chemerin rescued this effect, indicating that chemerin is required for osteoclast differentiation [135]. The chemerin/CMKLR1 signaling pathway has also been shown to inhibit β-catenin expression, subcellular localization, and transcriptional activity, thereby interfering with the Wnt pathway [136,137]. CMKLR1 is a Wnt-responsive gene, and activation of the Wnt pathway negatively regulates CMKLR1 expression, whereas upregulation of CMKLR1, in turn, suppresses β-catenin expression. The two pathways jointly regulate Wnt-mediated osteogenic differentiation and thereby maintain the balance between osteogenic and adipogenic differentiation of bone marrow mesenchymal stem cells [136]. In vitro experiments showed that siRNA-mediated knockdown of chemerin or CMKLR1 inhibited the osteogenic differentiation of BMSCs and promoted adipogenic differentiation [138]. However, *Cmklr1*^−/−^ mice exhibited reduced osteogenic capacity, and BMSCs derived from these mice also showed a significant reduction in osteogenic capacity [139].

#### Vascular Smooth Muscle Cells

**Figure 3:**
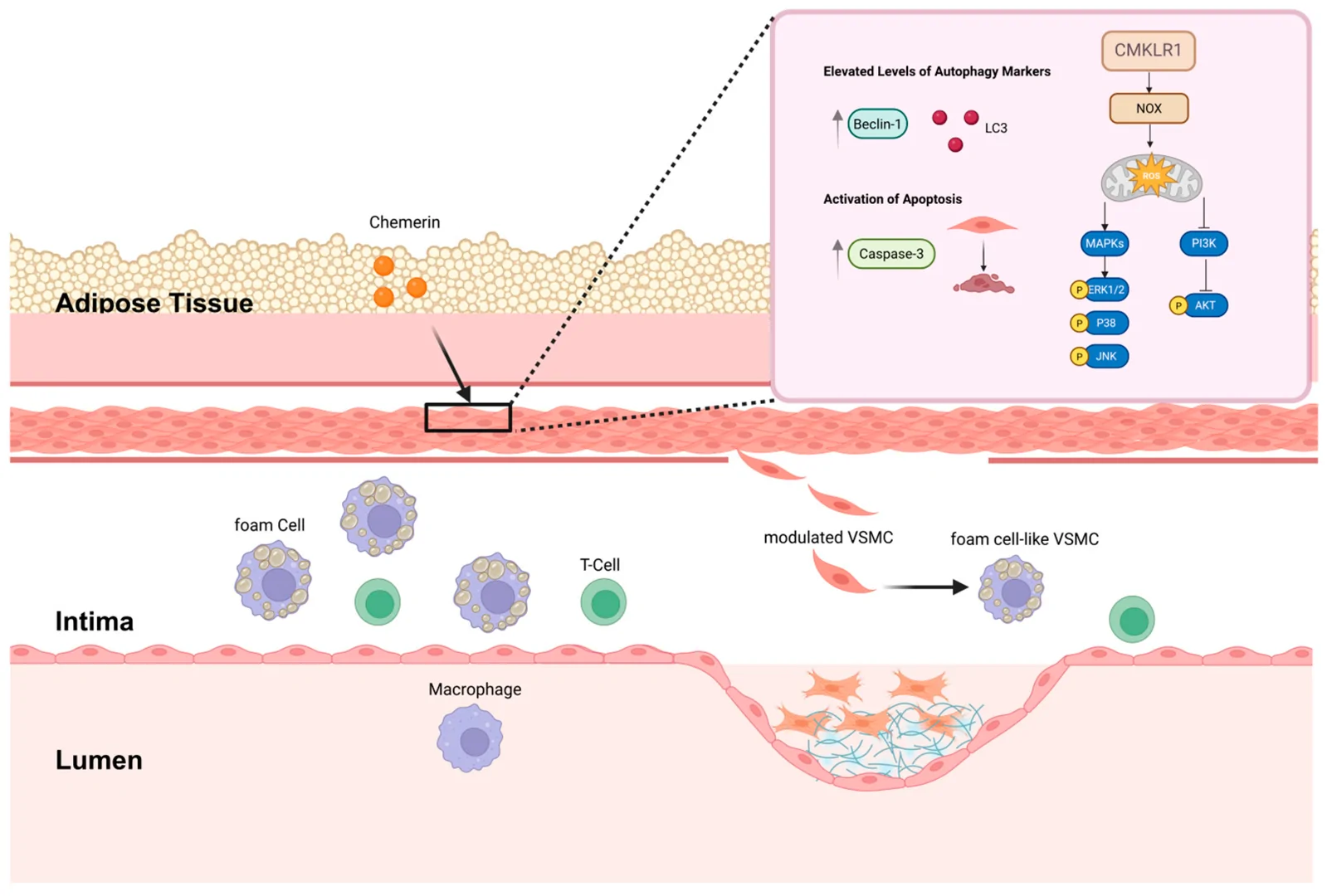
Role of paracrine-derived chemerin in regulating AS. Chemerin exerts pro-proliferative and pro-migratory effects on VSMCs through ROS-mediated activation of the MAPK signaling pathway and autophagy regulation. However, prolonged exposure to chemerin inhibits the PI3K/AKT signaling pathway, induces apoptosis, and exacerbates plaque instability.

Abnormal adipose accumulation surrounding organs, including PVAT and epicardial adipose tissue, is closely associated with multiple organ dysfunctions. The perivascular microenvironment is composed of local adipocytes and adjacent vascular cells. In vitro studies show that conditioned medium from adipocytes of obese mice contains elevated chemerin levels and significantly enhances oxidative stress and inflammatory responses in VSMCs [103]. These findings indicate that under inflammatory conditions, adipocytes undergo adaptive secretory changes that mediate complex intercellular signaling and regulate local vascular cell function. VSMCs exhibit high phenotypic plasticity in AS [140]. Emerging evidence suggests that foam cells are not exclusively macrophage-derived, as approximately 50% originate from VSMCs [141,142]. Cholesterol accumulation may lead to the downregulation of cholesterol efflux-related genes in VSMCs, promoting foam cell-like transformation [143]. VSMC proliferation is a response to vascular injury or inflammation. Under pathological conditions, VSMCs undergo dedifferentiation and migrate into the intima, resulting in intimal thickening. The pro-proliferative and pro-migratory effects of chemerin on VSMCs are closely associated with oxidative stress. (Figure 3) Chemerin activates MAPK signaling in a ROS-dependent manner [144] and participates in ROS-regulated autophagy processes [145], jointly promoting VSMC proliferation and migration.

However, the effects of chemerin on VSMCs are highly dependent on stimulation duration. In vitro studies indicate that prolonged exposure to chemerin inhibits the PI3K/Akt pathway and induces apoptosis [103,144,145], thereby exacerbating plaque instability and increasing susceptibility to rupture. Chemerin exerts pro-proliferative and pro-migratory effects on VSMCs through ROS-mediated activation of the MAPK signaling pathway and autophagy regulation. However, prolonged exposure to chemerin inhibits the PI3K/AKT signaling pathway, induces apoptosis, and exacerbates plaque instability. Nevertheless, existing studies cannot clearly distinguish the tissue source of chemerin and the effects of chemerin on VSMCs cannot exclude the contribution of systemic endocrine pathways.

## 7. Therapeutic Implications of Targeting Chemerin in AS

As research into the roles of inflammation, endothelial dysfunction, and lipid regulation in cardiovascular diseases continues to advance, the chemerin/CMKLR1 signaling pathway may play an important role and has emerged as a potential therapeutic target for AS. Several therapeutic strategies targeting the chemerin signaling pathway have been proposed. Statins can act directly on hepatocytes; in addition to reducing LDL-C, TC, and TG in patients with hypercholesterolemia [146], they can also reduce circulating chemerin and high-sensitivity C-reactive protein levels [147]. Another study showed that patients with CAD who received low-dose aspirin had lower circulating chemerin levels [148]. Combining chemerin-targeted therapies with existing treatments may therefore improve the efficacy of current therapeutic strategies. In addition, several inhibitors targeting the chemerin receptor CMKLR1 have been investigated. The small-molecule inhibitor CCX832 has been reported to be a highly selective CMKLR1 antagonist [149]. It was found to reduce vascular oxidative stress and significantly inhibit vasoconstriction induced by endogenous chemerin secreted from PVAT [150]. The addition of CCX832 significantly reduced the amplitude of EFS-induced contraction in vessels from WT rats, whereas no such effect was observed in vascular tissues from knockout rats [151]. 2-(α-Naphthoyl)ethyltrimethylammonium iodide (α-NETA) is another CMKLR1 inhibitor that can prevent chemerin-stimulated binding of CMKLR1 to β-arrestin [152]. α-NETA has also been reported to exert beneficial effects in the treatment of myocardial fibrosis [132] and obesity-related metabolic disorders [153]. Resolvin E1 (RvE1), a metabolite of eicosapentaenoic acid, is currently considered another ligand of CMKLR1. It exerts anti-inflammatory effects through CMKLR1 or Leukotriene B4 Receptor 1 [154]. RvE1 inhibits VSMC migration, reduces neutrophil infiltration into the vasculature, and promotes M2 macrophage polarization and the uptake and phagocytosis of oxLDL [155,156]. EPA supplementation has been suggested to alleviate Western diet-induced AS in *Apoe*^−/−^ mice by increasing the interaction between RvE1 and CMKLR1 [156]. Previous studies have also proposed that RvE1 may compete with chemerin for the same recognition site on CMKLR1 [157]. However, whether RvE1 exerts similar beneficial effects under physiological or pathological conditions without intervention remains unclear. Recently, an agonistic monoclonal antibody with RvE1-like properties and specificity for CMKLR1 has been developed. This antibody was shown to accelerate the resolution of acute inflammation and reduce tissue lesions and fibrosis in mice with inflammatory bowel disease [158]. Curcumin, a naturally occurring compound, has also been reported to inhibit VSMC proliferation and migration by suppressing the chemerin/CMKLR1/lipocalin 2 pathway, thereby delaying the progression of AS [159]. Existing studies have further shown that CMKLR1 knockdown is associated with the reversal of angiogenesis, reduced oxidative stress, and downregulation of autophagy-related gene expression, suggesting that CMKLR1 inhibitors may represent potential candidates for the treatment of AS. However, these approaches remain under investigation, and further studies are required to determine their safety and efficacy in the treatment of AS.

## 8. Limitations and Future Directions

Current evidence indicates that chemerin is closely associated with the initiation and progression of AS. Circulating chemerin levels may represent a potential target for intervention in hypertension, kidney disease, inflammatory diseases, and metabolic syndrome. Nevertheless, several limitations remain in current research on chemerin. First, measurements of chemerin forms are currently performed predominantly using commercial ELISAs to determine total chemerin levels. However, the sensitivity of these assays varies among different chemerin variants, and thus the quantitative results may be affected by the relative abundance of individual isoforms [17]. Isoform-specific ELISAs for human and mouse chemerin forms have been developed, but their application remains challenging because of the complex experimental procedures and the limited availability of corresponding antibodies. Although chemerin is predominantly secreted by the liver, adipose tissue, pancreas, adrenal glands, and certain specific cell types can also secrete chemerin.

Global knockout of chemerin has been shown to affect visceral adipose tissue formation without affecting subcutaneous adipose tissue [160]. This finding suggests that the effects of chemerin may differ among tissues. Indeed, different chemerin isoforms can be generated in different tissues [161]. Multiple animal studies (Table 1) and human studies (Table 2) have revealed that locally produced chemerin exerts important biological functions and differs from chemerin derived from systemic circulation.

In addition, current evidence regarding the expression of chemerin in tissues under pathological conditions remains inconsistent. Studies have reported increased, unchanged, and decreased hepatic chemerin mRNA levels in individuals with obesity [44,166,167,168]. Similar discrepancies have also been observed in animal studies. Chemerin expression in visceral adipose tissue was found to be decreased in *db/db* mice [169]. In contrast, chemerin expression in both visceral and subcutaneous adipose tissues was increased in obese and type 2 diabetic Psammomys obesus compared with normoglycemic animals [170]. Therefore, although chemerin has the potential to serve as a biomarker of cardiovascular disease, its application is limited by both technical constraints and the complexity of its underlying biological mechanisms. More precise detection methods need to be developed, together with a deeper understanding of its tissue-specific functions. Given the important role of chemerin in the initiation and progression of AS, clarifying the specific functions of chemerin derived from different tissues is essential for the development of targeted therapies for AS. To achieve this goal, future studies should develop tissue-specific *Rarres2* knockout or overexpression mouse models targeting tissues such as the liver and PVAT, determine how different forms of chemerin are generated and respond to physiological and pathological conditions, and distinguish the independent and synergistic effects of chemerin from different tissue sources during AS progression.

## 9. Conclusions

This review systematically focuses on the heterogeneity of tissue sources and distinguishes between endocrine and local paracrine pathways to summarize the differential roles of chemerin derived from different tissues in the initiation and progression of AS. Chemerin secreted by the liver and white adipose tissue primarily mediates systemic metabolic disturbances through endocrine pathways. Circulating chemerin mainly acts on vascular endothelial cells and macrophages, promoting progression of AS by inducing endothelial dysfunction, monocyte–endothelial adhesion, foam cell formation, and intraplaque angiogenesis. In contrast, chemerin produced by local PVAT and EAT exerts predominantly local paracrine effects, independent of elevated circulating concentrations. It directly acts on adjacent vascular smooth muscle cells to regulate vasoconstriction, smooth muscle cell proliferation, migration, and apoptosis, thereby exacerbating local vascular inflammation and plaque instability. Chemerin may therefore represent a potential therapeutic target for AS. Future studies should employ more tissue-specific knockout and overexpression animal models, combined with isoform-specific detection approaches, to clarify the independent functions and interactions of chemerin from different tissue sources and provide a theoretical basis for targeted interventions in ASCVD.

## Figures and Tables

**Table 1:** Animal studies on the tissue-specific effects of chemerin in AS.

Reference	Species	Sex	Disease	AS-Associated Conclusions	Tissue and Chemerin Levels
[62]	Liver ASO, Dahl salt-sensitive rats	Male	Hypertension	Most circulating chemerin is secreted by the liver. Chemerin involved in blood pressure regulation may originate primarily from local tissues rather than systemic circulation.	Circulation and liver ↓ Retroperitoneal fat and mesenteric perivascular adipose tissue ⟷
[162]	*Rarres2*^−^^/−^, Sprague Dawley rats	Male & Female	Hypertension	Chemerin expression has sex differences. Chemerin is able to modify blood pressure in response to a hypertensive challenge.	WT: Female < Male (circulating and tissue chemerin levels) KO: Circulation, liver, and retroperitoneal fat ↓
[127]	Dahl salt-sensitive rats	Male & Female	Hypertension	Chemerin expression exhibits sex differences. PVAT is a major source of bioactive chemerin.	PVAT: Female < Male
[163]	NOS3^fl/fl^Adipoq-Cre^ERT2^, C57BL/6J	No data	Obesity	Chemerin may be regulated by adipocyte NOS3 and may contribute to obesity-induced vascular dysfunction.	Circulation, PVAT, mesenteric perivascular adipose tissue ↑
[69]	AAV9-TBG-sg*Rarres2*, C57BL/6J	Male	Obesity	Liver-derived chemerin may mediate hyperlipidemia, liver injury, and systemic inflammation.	Liver ↓
[56]	*Apoe*^−/−^, C57BL/6J; *Ccrl2*^−/−^ *Apoe*^−/−^, C57BL/6J; HUVECs; THP-1	Male	AS	Disturbed blood flow induces CCRL2 expression, which recruits chemerin and promotes AS by activating β2 integrins on monocytes.	Aorta: chemerin ↑ at 4 weeks of HFD, returning to baseline at 12 weeks ↑ in regions of disturbed blood flow.

↓ decreased; ⟷ no change; ↑ increased.

**Table 2:** Human studies on the tissue-specific effects of chemerin in AS.

Reference	Number	Sex	Disease	AS-Associated Conclusions	Tissue And Chemerin Levels
[64]	45	Male & Female	Liver cirrhosis	The liver is a major source of circulating chemerin.	Portal vein ≈ peripheral vein; hepatic vein > portal vein.
[164]	3986	Male & Female	/	The association of chemerin with inflammation and metabolic syndrome cannot be attributed solely to adipose tissue accumulation.	Circulating chemerin: Female > Male VAT and SAT were positively associated with circulating chemerin, with a stronger association for VAT.
[165]	47	Male & Female	Chronic Kidney Disease	Chemerin may regulate vascular function through an indirect, liver-mediated pathway.	Circulating chemerin levels were significantly associated with hepatic fetuin-A, a calcification inhibitor.
[113]	40	Male & Female	Hypertension/Acute Coronary Occlusion	Locally produced chemerin contributes to the progression of atherosclerotic lesions.	Chemerin immunoreactivity in PVAT and foam cells was significantly correlated with atherosclerotic lesion severity in both the aorta and coronary arteries.
[128]	100	Male & Female	Coronary Artery Disease	Epicardial adipose tissue may secrete chemerin and contribute to vascular remodeling.	Circulating chemerin and VEGF ↑ with epicardial adipose tissue volume Circulating chemerin, VEGF, and epicardial adipose tissue volume were independent risk factors for vascular remodeling.
[131]	53	Male & Female	Coronary Artery Disease	Chemerin secreted by epicardial adipose tissue may contribute to the progression of AS.	Epicardial adipose tissue chemerin: positively correlated with Gensini score Circulating chemerin: not associated with Gensini score.

≈ approximately; > higher than; ↑ increased.

## Data Availability

No new data were created or analyzed in this study. Data sharing is not applicable to this article.
